# Supplementary material for: A New Flavanone from Chromolaena tacotana (Klatt) R. M. King and H. Rob, Promotes Apoptosis in Human Breast Cancer Cells by Downregulating Antiapoptotic Proteins
Source: Molecules. 2022 Dec 21;28(1):58. doi: 10.3390/molecules28010058 (PMC9822081; doi:10.3390/molecules28010058)
Supplement: Supplementary file 1 [file molecules-28-00058-s001.zip › molecules-2057939-supplementary.pdf]

SUPPLEMENTARY INFORMATION

Table S1. NMR data and C-H correlations for the Flavanone Tacotanina.

CARBON	δ ^{13}C(ppm)	APT	HSQC δ^{1}H- δ^{13}C (ppm)	HMBC -δ^{1}H- δ^{13}C (ppm)
C-2	78.78	(-) CH	5.34-78.78	5.34-114.65;118.28,130.17;188.59
C-3	45.20	(+) CH_2	2.58,298-45.20	2.58,2.98-78.78;130.17; 164.83; 188.59
C-4	188.59	(+) C		
C-5	162.18	(+) C		3.83-162.18
C-6	93.16	(-) CH	6.17-93.47	6.17-93.16;94.09;105.79
C-7	165.79	(+) C		
C-8	94.09	(-) CH	6.19-94.09	6.19-94.09;105.79;162.18;165.79;188.59
C-9	164.92	(+) C		
C-10	105.79	(+) C		
C-1′	130.17	(+) C		
C-2′	114.65	(-) CH	6.88-114.65	6.88-78.78;118.28;130.17;145.61
C-3′	145.61	(+) C		
C-4′	146.03	(+) C		
C-5′	118.30	(-) CH		
C-6′	115.75	(-) CH		
C5-OCH3	56.15	(+) CH_3		3.83-162.18
C7-OCH3	56.27	(+) CH_3		3.87-165.79

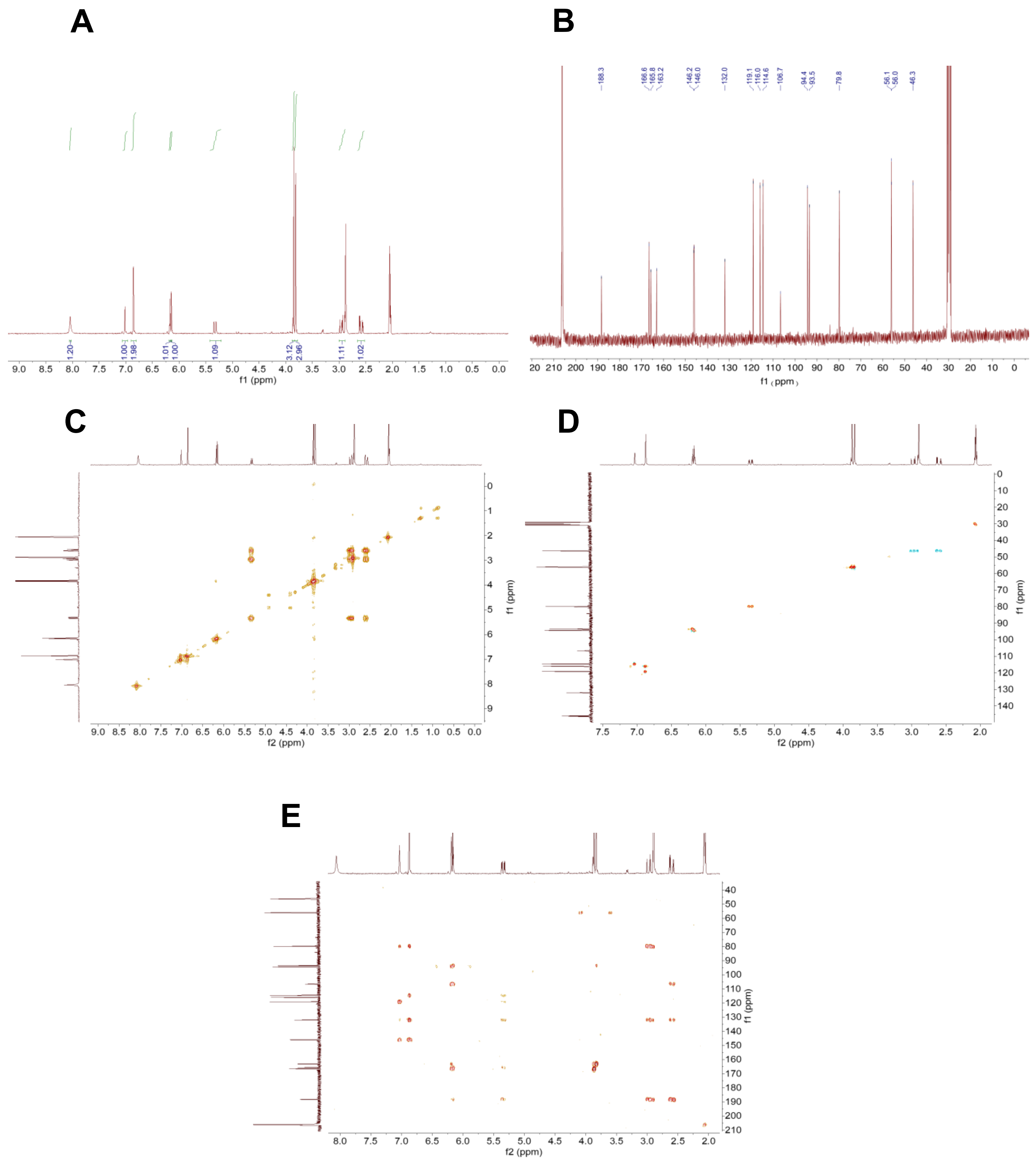

Figure S1. Nuclear Magnetic Resonance spectroscopy (300 MHz, Acetone-d_6) of compound tacotanina. **A.** ^{1}H NMR spectrum. **B.** ^{13}C NMR spectrum (75 MHz, $CDCl_3$). **C.** ^{1}H-^{1}H COSY spectrum. **D.** HSQC spectrum. **E.** HMBC spectrum.

A

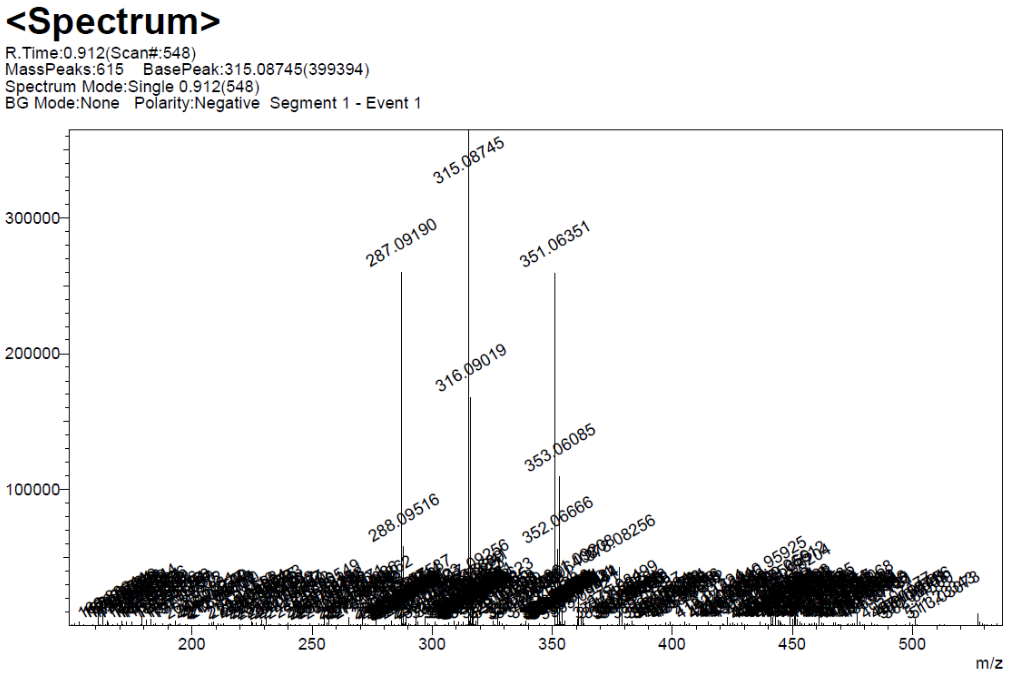

B

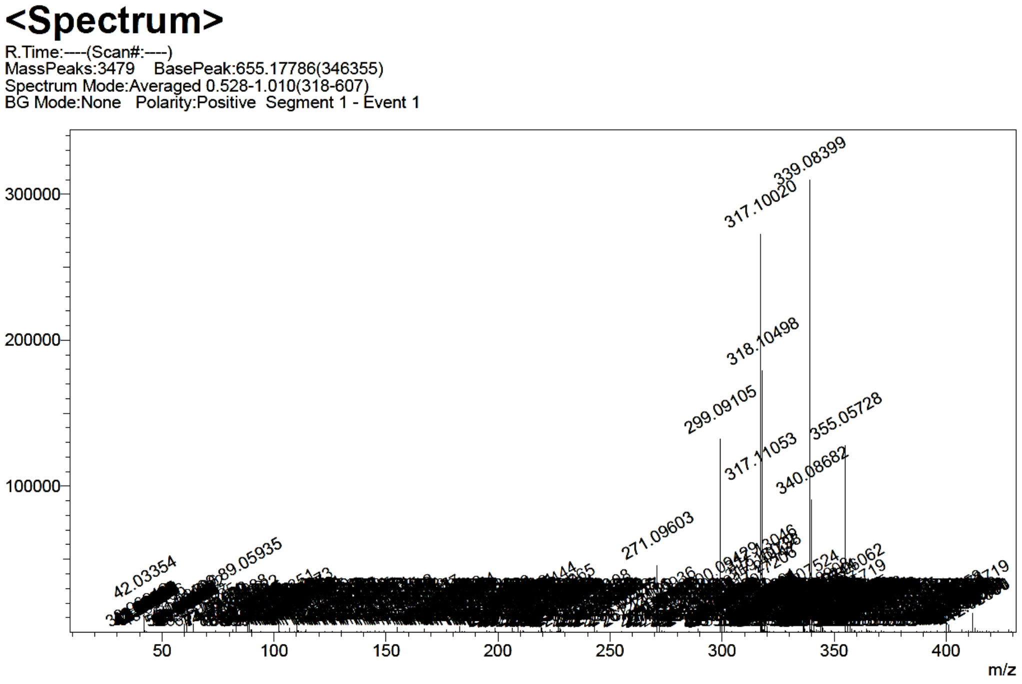

Figure S2. HPLC/QTOF analysis A) ESI negative ion mode, $[M-H]^-$: 315,08745, and B) positive ion mode $[M+H]^+$: 317,10020.

A

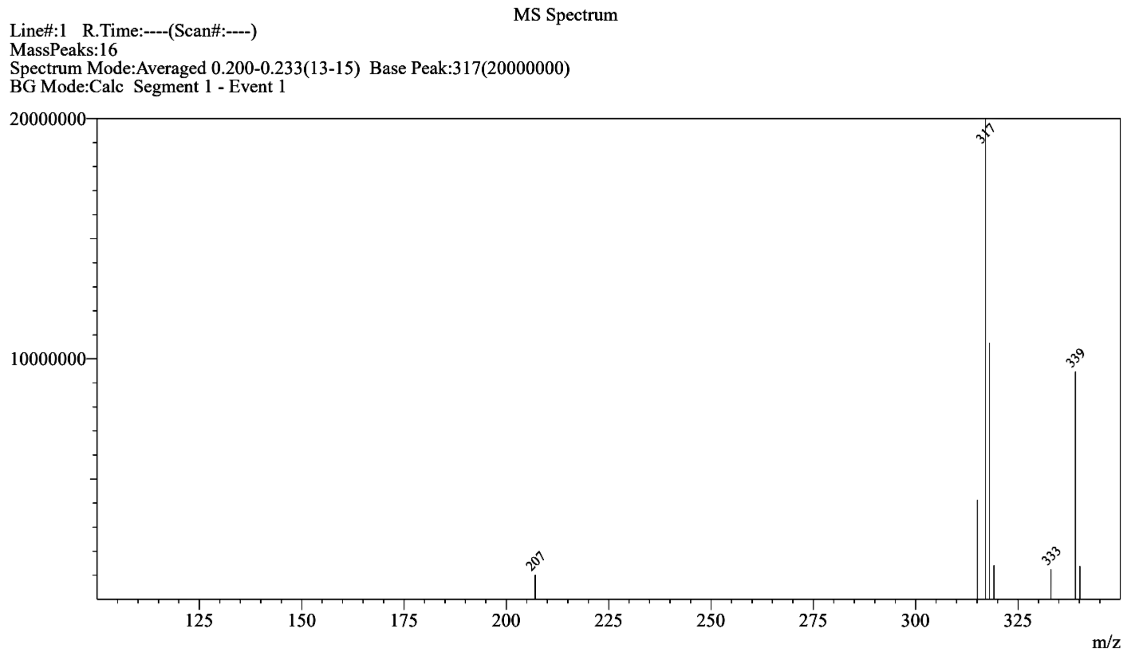
MS Spectrum
Line#:1 R.Time:----(Scan#:----)
MassPeaks:16
Spectrum Mode:Averaged 0.200-0.233(13-15) Base Peak:317(20000000)
BG Mode:Calc Segment 1 - Event 1
20000000
10000000
317
339
207
333
125
150
175
200
225
250
275
300
325
m/z

B

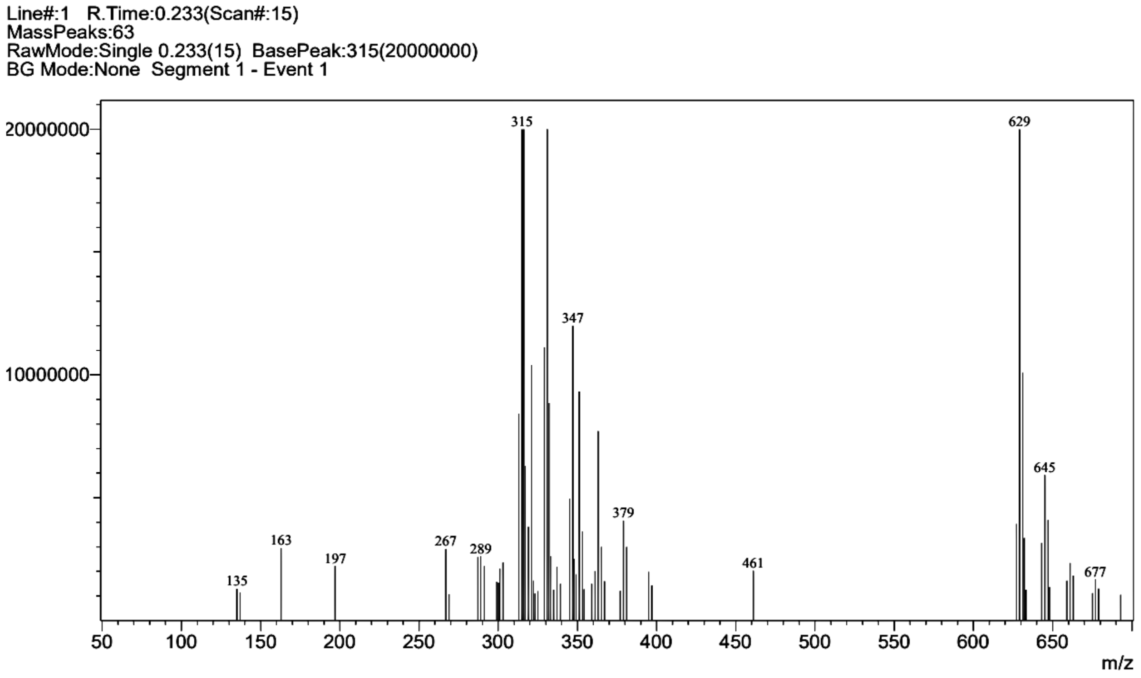
Line#:1 R.Time:0.233(Scan#:15)
MassPeaks:63
RawMode:Single 0.233(15) BasePeak:315(20000000)
BG Mode:None Segment 1 - Event 1
20000000
10000000
315
629
347
645
379
135
163
197
267
289
461
677
50
100
150
200
250
300
350
400
450
500
550
600
650
m/z

C

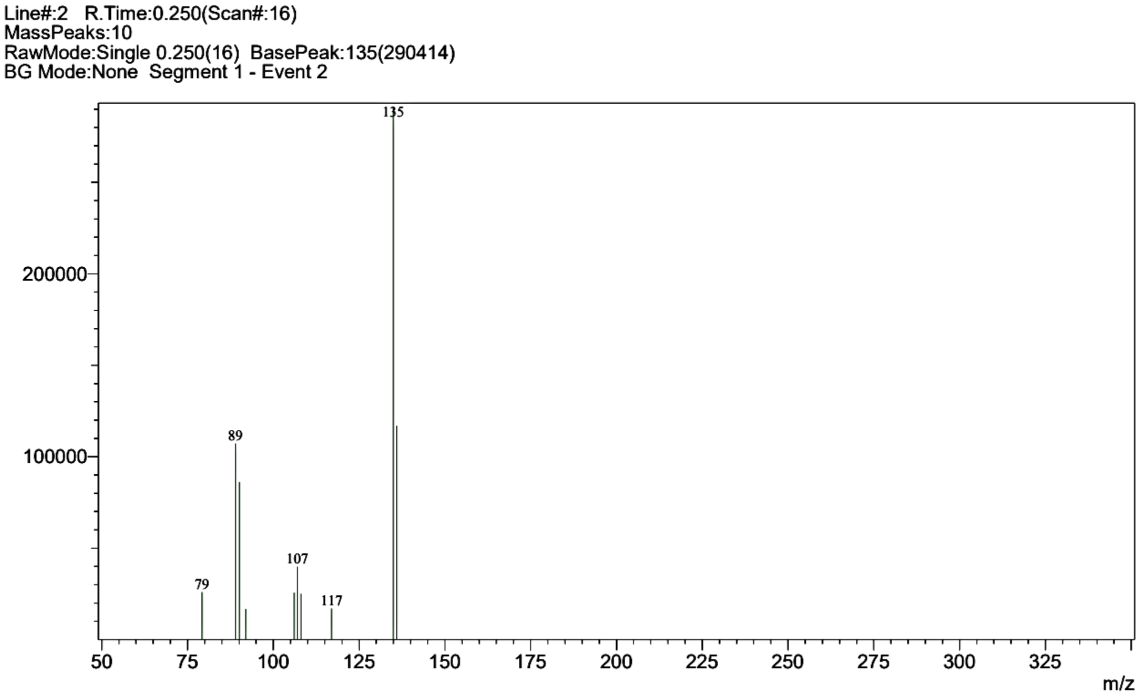

Figure S3. HPLC/MS analysis A) ESI positive ion mode, $[M + H]^+$: 317, and B) ESI negative ion mode $[M - H]^-$: 315, and C) MS^2 of the ion m/z 135.
